# Metal‐Free N, P‐Codoped Carbon for Syngas Production with Tunable Composition via CO_2_ Electrolysis: Addressing the Competition Between CO_2_ Reduction and H_2_ Evolution

**DOI:** 10.1002/cssc.202402249

**Published:** 2025-01-03

**Authors:** Ryuji Takada, Hiroyuki Okada, Kotaro Narimatsu, Koji Miyake, Yoshiaki Uchida, Etsushi Tsuji, Norikazu Nishiyama

**Affiliations:** ^1^ Division of Chemical Engineering Department of Materials Engineering Science Graduate School of Engineering Science Osaka University 1-3 Machikaneyama Toyonaka Osaka 560-8531 Japan; ^2^ Center for Research on Green Sustainable Chemistry Tottori University 4-101 Koyama, Tottori Tottori 680-0945 Japan; ^3^ Innovative Catalysis Science Division Institute for Open and Transdisciplinary Research Initiatives (ICS-OTRI) Osaka University Suita, Osaka 565-0871 Japan

**Keywords:** N and P co-doped carbon, CO_2_electroreduction, Metal-free catalyst, Syngas, Density functional theory calculation

## Abstract

Electroreduction of carbon dioxide into value‐added fine chemicals is a promising technique to realize the carbon cycle. Recently, metal‐free heteroatom doped carbons are proposed as promising cost‐effective electrocatalysts for CO_2_ reduction reaction (CO_2_RR). However, the lack of understanding of the active site prevents the realization of a high‐performance electrocatalyst for the CO_2_RR. Herein, we synthesized metal‐free N, P co‐doped carbons (NPCs) for producing syngas, which is composed of H_2_ and CO, by CO_2_ electrolysis using inexpensive bio‐based raw materials via simple pyrolysis. The syngas ratio (H_2_/CO) can be controlled within the high demand range (0.3–4) at low potentials using NPCs by tuning the N and P contents. In comparison with only N doping or P doping, N and P co‐doping has a positive impact on improving CO_2_RR activity. Experimental analysis and density functional theory (DFT) calculations revealed that negatively charged C atoms adjacent to N and P atoms are the most favorable active sites for CO_2_‐to‐CO conversion compared to pyridinic N on N, P co‐doped carbon. Introducing N atoms generates the preferable CO_2_ adsorption site, and P atoms contribute to decreasing the Gibbs free energy barrier for key *COOH intermediates adsorbed on the negatively charged C atoms.

## Graphical abstract

We synthesize metal‐free N, P co‐doped carbons (NPCs) derived from inexpensive bio‐based raw materials via simple pyrolysis. The syngas ratio (H_2_/CO) can be controlled within the high demand range (0.3–4) by using NPCs by tuning the raw material compositions. Experimental analysis and density functional theory (DFT) calculation revealed that negatively charged C atoms adjacent to N and P atoms are the most favorable active sites for CO_2_‐to‐CO conversion on N, P co‐doped carbon. The presence of P atoms contributes to decreasing the free energy barrier for key *COOH intermediate adsorption.

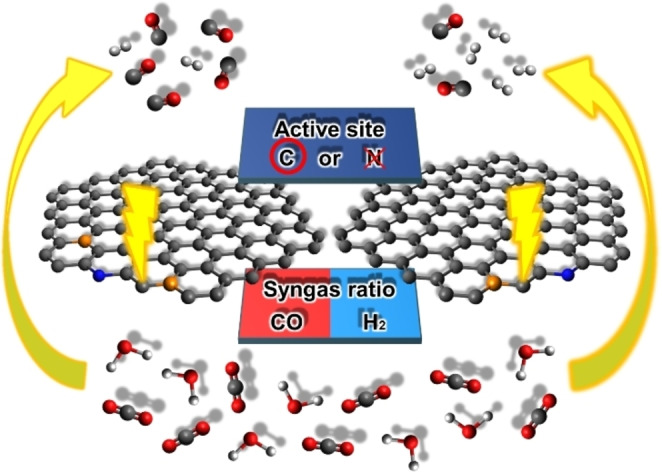

## Introduction

With the excessive utilization of fossil fuels, increasing anthropogenic CO_2_ emissions have resulted in serious global warming and climate change issues.[^1^, ^2^] Alternative power resources for fossil fuels and effective CO_2_ conversion systems are urgently required to mitigate these issues. The electrochemical carbon dioxide reduction reaction (CO_2_RR) driven by renewable energy has been regarded as a promising and sustainable strategy to reduce CO_2_ emissions and convert the CO_2_ into highly valued commodity chemicals.[^3^, ^4^, ^5^] Among these chemicals, much attention has been paid to CO_2_ electroreduction into CO, because the mixture of CO and H_2_, syngas, can be utilized for the existing thermochemical processes. In the practical processes, the specific ratio of syngas is required for downstream application.[^6^, ^7^, ^8^] For example, the syngas ratio (H_2_/CO) of 2.0 or 0.3–4.0 can be applied to methanol synthesis or Fischer Tropsch (FTS) synthesis, respectively.[^9^, ^10^, ^11^] Conventionally, the syngas is produced through the steam reforming of natural gas or the gasification of coal under the severe conditions, furthermore, this conventional syngas production cannot control syngas proportion precisely.[^12^, ^13^] From this aspect, CO_2_ electroreduction into CO with the assistance of H_2_ production via water electrolysis is advantageous in the one‐step synthesis of syngas under mild conditions. To date, metallic electrocatalysts have been extensively explored to obtain syngas via CO_2_RR. However, their high cost, possible pollution of the environment, and poor durability hinder the large‐scale application of CO_2_RR.[^14^, ^15^, ^16^] Therefore, low‐cost, environmentally friendly, and highly stable electrocatalysts that can generate syngas and control its proportion are highly desirable.

Metal‐free carbon materials doped with heteroatoms (such as N, B, P, S) have been reported as electrocatalysts for CO_2_RR alternatives to metallic catalysts.[^17^, ^18^, ^19^] The catalytic activity is most likely derived from charge redistribution by the introduction of heteroatoms. However, there are many cases that heteroatom doped carbon materials are not cost‐effective catalysts due to the use of expensive raw materials and complicated synthesis method. Recently, metal‐free N, P co‐doped carbon materials have been developed to further enhance the catalytic performance. C. Chen et al. and Y. Yan et al. have developed N, P co‐doped carbon aerogels, which produce CO with high selectivity.[^20^, ^21^] These studies have shown that pyridinic N is a possible active site. On the other hand, J. Xie et al. have reported that C atom is the possible active site for CO_2_ reduction into CO on N, P co‐doped carbon material.^[22]^ As described, the active sites for CO_2_RR on N, P co‐doped carbons have not been elucidated, because the active sites are revealed only by theoretical calculations. To gain a full understanding of the active sites, both experimental and theoretical approaches are necessary.

In this work, we report a facile synthesis of metal‐free N, P co‐doped carbons with various N and P contents, which can control the syngas ratio. These catalysts were easily prepared via one‐step pyrolysis using fully bio‐based glycine and phytic acid.^[23]^ The active sites for CO_2_‐to‐CO conversion and the key factors that determine the generation of CO or H_2_ were investigated by experimental analysis and density functional theory (DFT) calculations by Gaussian. This work provides the rational design of N, P co‐doped carbon, which can control the syngas ratio within the high demand range. To the best of our knowledge, this is the first study showing that negatively charged C atoms adjacent to N and P atoms can be active sites for CO_2_‐to‐CO conversion on N, P co‐doped carbon. In addition, only a few studies have performed DFT calculations using Gaussian; thus, this study also provides a theoretical approach to reveal the reaction mechanism using Gaussian.

## Results and Discussion

**Figure 1:**
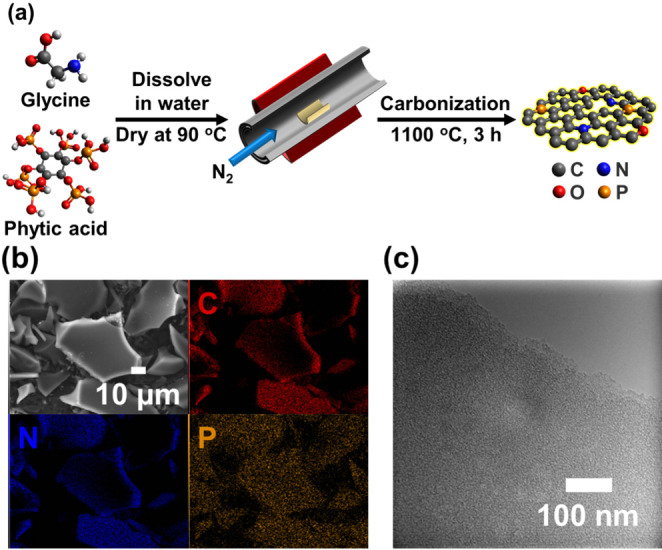
a) Scheme illustrating the synthesis of NPCs. b) Corresponding elemental mapping of NPC‐1. c) TEM image of NPC‐1.

N, P co‐doped carbons were synthesized by carbonizing the mixture of glycine and phytic acid in a mass ratio of 1: *x* (*x*=0.5, 1, 2) for 3 h at 1100 °C under N_2_ atmosphere to explore the effects of different N and P contents on the catalytic activity for CO_2_RR (Figure 1a). The obtained catalysts were denoted as NPC‐*x*. To investigate the effect of N and P co‐doping, only glycine or phytic acid were carbonized under the same conditions. The obtained N or P doped carbon were denoted as NC or PC, respectively. As shown in Figure 1b and Figure S1, scanning electron microscopy‐energy dispersive X‐ray spectroscopy (SEM‐EDX) confirmed the presence of C, N and P and the uniform distribution of N and P atoms on the NPCs, indicating successful N and P co‐doping. The C, N, P, and O contents of the synthesized catalysts were explored by EDX and CHN analysis (Table S1). The N and P contents in the NPCs varied according to the raw material compositions. The morphologies of the catalysts were investigated by transmission electron microscopy (TEM). As displayed in Figure 1c and Figure S2, the sheet structures were observed for all the catalysts and the pore structures were observed for NPCs and PC.

**Figure 2:**
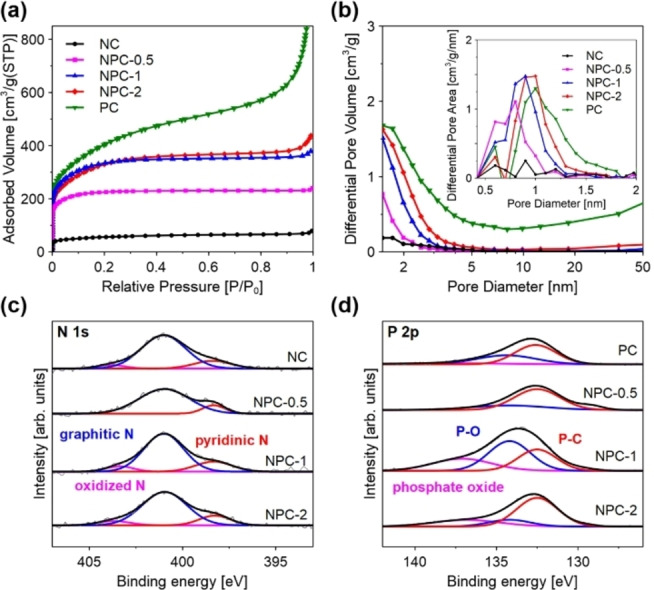
a) N_2_ adsorption isotherms, and b) pore size distributions of each catalyst. High‐resolution XPS spectra of each catalyst: c) N 1s, and d) P 2p.

The N_2_ adsorption measurements were conducted to further investigate the pore structures of each catalyst. N_2_ adsorption isotherms and pore size distributions confirmed that NPCs have a micro‐ and mesoporous structure, while PC displayed micro‐, meso‐, and macroporous structures (Figures 2a and b). NC showed less pore structure, with a low Brunauer‐Emmett‐Teller (BET) specific surface area and pore volume (Table S2). The BET surface areas of NPCs gradually increased by the amount of phytic acid used as a raw material because phytic acid functioned as an activator, such as H_3_PO_4_ activation.[^24^, ^25^]

The graphitic nature and defects related to heteroatoms doping were investigated by performing X‐ray diffraction (XRD) and Raman spectra. As shown in Figure S3, two broad diffraction peaks centered at 24.1° and 44.1° were observed in all the catalysts, corresponding to the (002) and (100) planes of carbon, confirming the crystalline nature of catalysts.[^26^, ^27^] As displayed in Figure S4, two intense peaks around 1345 cm^−1^ and 1580 cm^−1^ associated with the D and G bands, respectively, were observed in the Raman spectra. NPCs and PC showed higher values of the *I*_D_/*I*_G_ ratio (NPC‐0.5:0.90, NPC‐1:0.91, NPC‐2:0.91, PC: 0.95) than NC (0.89), suggesting that the number of defects increased by P doping because of the larger atomic size of P atoms relative to that of C atoms. X‐ray photoelectron spectroscopy (XPS) measurements were performed to study the bonding states of the catalyst surfaces. As shown in Figure 2c, the N 1s spectra were fitted into three peaks, representing pyridinic N (~398.4 eV), graphitic N (~401.0 eV), and oxidized N (~403.8 eV).[^28^, ^29^, ^30^] The P 2p spectra can be divided into three peaks (Figure 2d), including P−C (~132.6 eV), P−O (~134.4 eV), and phosphate oxide (~138.0 eV).[^31^, ^32^, ^33^] N and P atoms introduced into carbon matrix could change the charge distribution and electronic state of the catalytic active sites. The contents of each N and P species are shown in Figure S5. In summary, the successful synthesis of N, P co‐doped carbons with high specific surface areas, large pore volumes, and abundant defects was concluded, which is expected to play a role in electrolysis.

**Figure 3:**
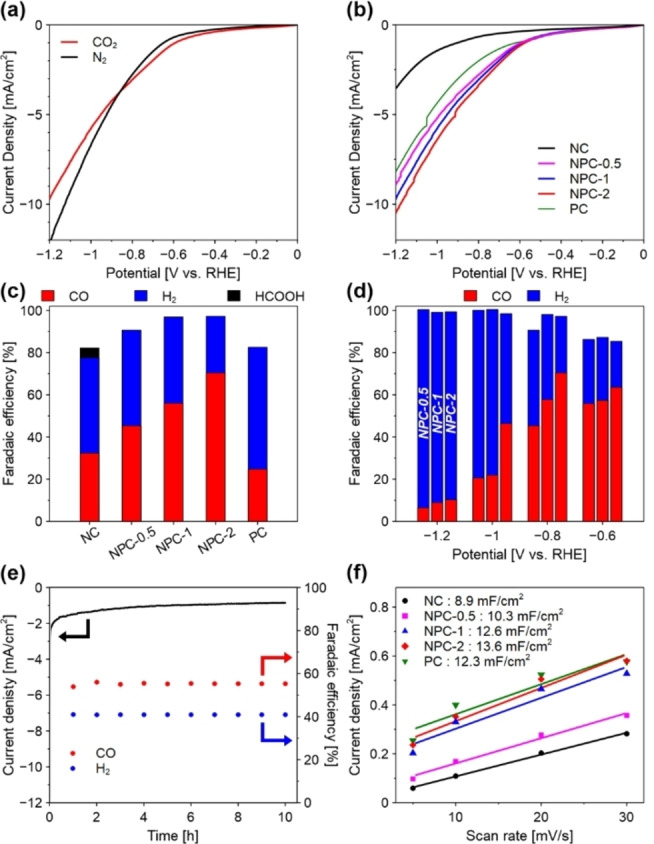
a) LSV curves in CO_2_‐saturated and N_2_‐saturated 0.1 M KHCO_3_ solution using NPC‐1 as an electrocatalyst. b) LSV curves in CO_2_‐saturated 0.1 M KHCO_3_ solution using each catalyst. c) Faradaic efficiencies of CO, H_2,_ and HCOOH at −0.8 V vs. RHE. d) Faradaic efficiencies of CO and H_2_ using NPCs within the potential window from −0.8 V to −1.2 V vs. RHE in CO_2_‐saturated 0.1 M KHCO_3_ solution. e) Chronoamperometric responses and faradaic efficiency of CO and H_2_ on NPC‐1 at −0.8 V vs. RHE for 10 h in CO_2_‐saturated 0.1 M KHCO_3_ solution. f) Double‐layer capacitance values of each catalyst.

In this context, the electrocatalytic activity of the CO_2_RR was evaluated by electrochemical measurements in an H‐type cell with a three‐electrode configuration. First, linear sweep voltammetry (LSV) measurements were conducted in CO_2_‐saturated and N_2_‐saturated 0.1 M KHCO_3_ solution. Taking NPC‐1 as an example, NPC‐1 showed a higher current density in CO_2_ than that in N_2_ at a potential positive than −0.8 V vs. reversible hydrogen electrode (RHE), implying high catalytic activity for CO_2_RR (Figure 3a). On the other hand, a higher current density was obtained in N_2_ than in CO_2_ at a negative potential than −0.8 V vs. RHE, suggesting the domination of hydrogen evolution reaction (HER). As shown in Figure 3b, the current density of NPCs and PC was superior to that of NC, implying that the larger S_BET_ and pore volume provide rich mass transportation and accessible active sites. To analyze the electroreduction products and selectivity of these products, the controlled potential electrolysis method was performed on each catalyst at −0.8 V vs. RHE for 2 h. Possible liquid products were identified using high‐performance liquid chromatography (HPLC). No liquid products were observed on the NPCs and PC, while a small amount of HCOOH was observed on the NC. Furthermore, the gas products were quantified by gas chromatography (GC). Only CO and H_2_ were detected on all the catalysts. As shown in Figure 3c and Table S3, the Faradaic efficiency of CO (FE_CO_) for NPCs was higher than that of NC and PC, indicating that N and P co‐doping could improve CO_2_RR performance more than N or P doping. NPC‐2 exhibited the highest FE_CO_ of 70.4 %, which was consistent with the result of LSV measurements (Figure 3b).

The selectivity of the reduction products for NPCs under various potentials was investigated using the controlled potential electrolysis method (Figure 3d and Table S4). Only gas products, mainly CO and H_2_, were detected within the potential window from −0.6 V to −1.2 V vs. RHE. A small amount of CH_4_ was detected on NPC‐1 at −1.2 V vs. RHE and NPC‐2 at −1.0 V and −1.2 V vs. RHE. The FE_CO_ of NPCs was relatively higher at low potentials, suggesting that NPCs promoted the CO_2_RR and suppressed the HER. Whereas the FE_CO_ decreased gradually as the applied potential decreased (less than −1.0 V vs. RHE), which means the domination of HER. At the potential range from −0.6 V to −1.0 V vs. RHE, syngas in the ratio of 0.3–4.0 (H_2_/CO) was obtained, which can be applied to Fischer‐Tropsch synthesis.[^9^, ^10^] When comparing the FE_CO_ at the same potential, the FE_CO_ of NPC‐2 was the highest and that of NPC‐0.5 was the lowest among the NPCs, implying that doping with more P atoms enhances the CO_2_RR activity. To confirm the carbon source of products by CO_2_RR, the electrolysis experiment was carried out on NPC‐1 in N_2_‐saturated electrolyte at −0.8 V vs. RHE (Figure S6). Only H_2_ was detected, suggesting that the CO_2_ gas dissolved in the electrolyte was the only carbon source for producing CO.

In addition to selectivity, the stability of the electrocatalyst for syngas production is an important factor for practical applications. The chronoamperometric response was performed on NPC‐1 at −0.8 V vs. RHE for 10 h (Figure 3e). There was a slight decay in the current density under continuous operation for up to 10 h due to the catalyst stripping from carbon paper and coating the catalyst surface with gas products. The FE for producing syngas remained stable. The elemental mapping of NPC‐1 after a long‐term test showed that C, N, and P atoms were still uniformly distributed (Figure S7a). In addition, no obvious changes in morphology, structure, and bonding state were observed (Figure S7b–d). As displayed in Figure S7d, the peak for phosphate oxide was not observed because phosphate oxide is soluble in electrolyte. From these results, it is believed that the structure of the active site did not change after a long‐term test, indicating the high stability of electrocatalyst.

To clarify the synergistic effects of introducing N and P atoms by experimental analysis, N doped carbon which showed the high BET surface area and pore volume was synthesized from glycine via carbonization and CO_2_ activation.[^34^, ^35^] The obtained porous N doped carbon is denoted as NC‐act, and a detailed description of the synthesis method is provided in the Supporting Information. The pore structure and N species content of NC‐act are presented in Table S2 and Figure S5a. The potential controlled electrolysis was also performed on NC‐act at −0.8 V vs. RHE. CO and H_2_ were detected, and the liquid products were below the detection limit. The FE_CO_ of NC‐act was much higher than that of NC, suggesting that the pore structure is a key factor in catalytic performance (Table S3). Although NC‐act showed a high BET surface area and pore volume similar to NPC‐2, the FE_CO_ of NC‐act was lower than that of NPC‐2. Therefore, it is expected that heteroatom doping had a more significant effect on activity than pore structure.

To verify this, the electrochemical surface area (ECSA) was estimated by measuring the double‐layer capacitance derived from cyclic voltammetry at various scan rates (Figure S8). As displayed in Figure 3f and Figure S9, NPC‐2 showed the largest ECSA in all the catalysts. Furthermore, the partial current density of CO (*j*_CO_) at −0.8 V vs. RHE normalized by ECSA was calculated (Figure S10). The normalized *j*_CO_ of NPCs was higher than that of NC‐act. Regarding the active species on N doped carbon, some studies have demonstrated that pyridinic N is the most favorable active site.[^36^, ^37^] As shown in Figure S5, NC‐act showed a much higher pyridinic N content than NPCs. However, the catalytic activity for CO_2_‐to‐CO conversion of NPCs is superior to that of NC‐act, which indicates that the active sites on N, P co‐doped carbons are different from those of N doped carbons. Furthermore, the activity for CO_2_‐to‐CO conversion of NC‐act was higher than that of PC, suggesting that the preferable active sites can be generated by introduced N species. This is also confirmed by electrochemical measurement for NPC‐3 (synthesized from glycine and phytic acid in a mass ratio of 1: 3), which showed lower FE_CO_ than NPC‐2 (Figure S11). In terms of the role of introducing P atoms, a significant difference of catalytic activity can be found among NPCs. Therefore, it is considered that the N and P contents had an influence on the CO_2_RR activity.

**Figure 4:**
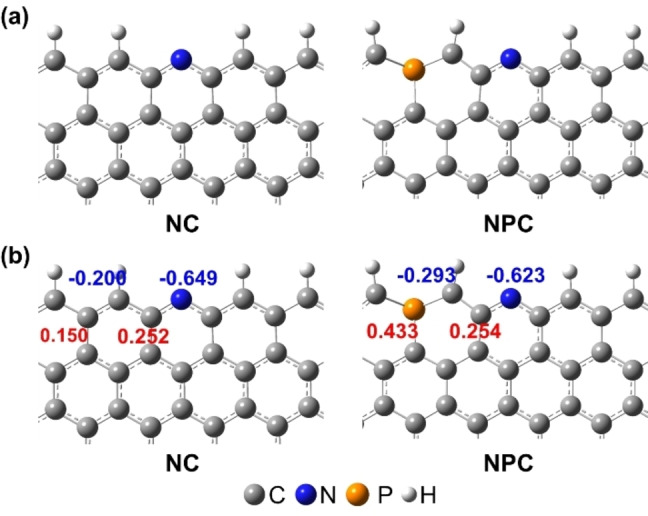
a) The computational structure model of N doped carbon and N, P co‐doped carbon configurations. b) The charge densities of computational structure models.

DFT calculations using Gaussian were performed to gain further insights into the CO_2_RR catalytic mechanisms on N, P co‐doped carbon. The introduction of N and P atoms into carbon matrix could modify the charge delocalization and electronic state of the active sites. Two computational structure models, which contain the possible active sites with pyridinic N and P−C, were constructed from the result of elemental and XPS analysis (Figure 4a and Figure S12). N doped carbon, and N, P co‐doped carbon configurations were denoted NC, and NPC, respectively. P atoms introduced into the carbon lattice mainly locate the edge sites due to their large atomic sizes relative to C atoms.[^21^, ^38^] The elemental steps of the electroreduction of CO_2_ into CO are provided in the Supporting Information. To clarify the relationship between charge density and CO_2_ adsorption sites, the population was calculated for each model (Figure 4b). The active sites were determined by searching for the lowest free energy of *COOH adsorption among all the possible active sites with pyridinic N and C atoms adjacent to N and P atoms, which demonstrates that pyridinic N and negatively charged C atoms were expected to be the active sites. In fact, CO_2_ molecules are considered to adsorb easily on a negatively charged site because C atom contained in CO_2_ molecule is positively charged.

**Figure 5:**
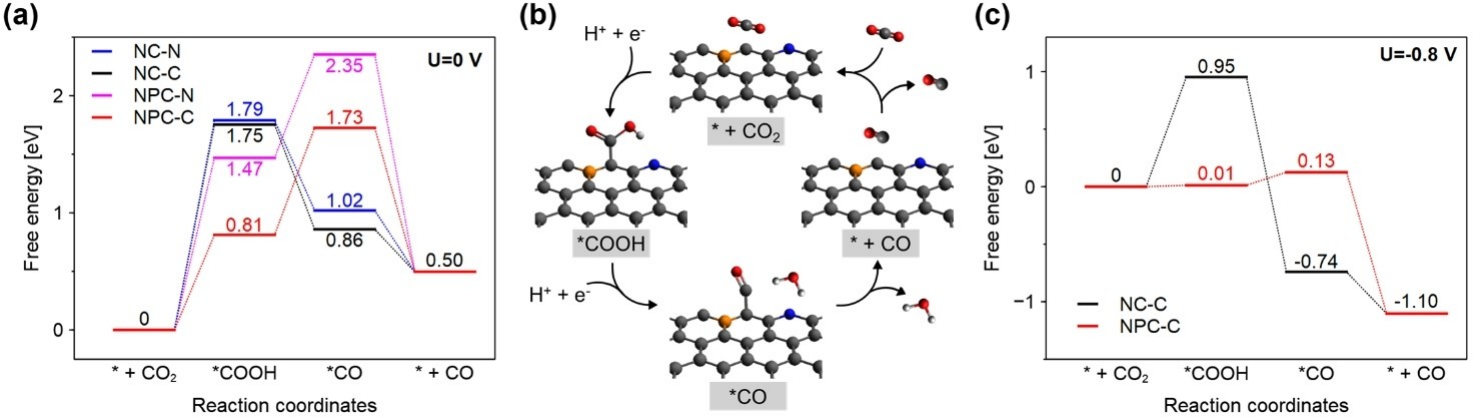
a) Gibbs free energy diagrams for CO_2_RR to CO on model NC or NPC. U=0 V. b) Reaction pathways for CO_2_RR to CO on N, P co‐doped carbon. The white, gray, blue, red, and orange spheres represent H, C, N, O, and P atoms, respectively. c) Gibbs free energy diagrams for CO_2_RR to CO on model NC−C and NPC−C. U=−0.8 V.

The corresponding Gibbs free energy diagrams and reaction pathways for CO_2_RR to CO are shown in Figure 5a and S13. −N and −C represent the case where pyridinic N and negatively charged C atoms are employed as an active site, respectively. The free energy diagrams were constructed at 0 V vs. RHE according to the computational hydrogen electrode (CHE) model.^[39]^ In terms of model NC, significant changes in Gibbs free energy were not observed no matter if pyridinic N or negatively charged C atom are employed as the active site. However, there were notable changes in the free energy diagram of the NPC model. The free energy barrier when employing the C atom as an active site was much lower than that when employing the N atom as an active site, which reveals that a negatively charged C atom can be a more favorable active site rather than pyridinic N. This is consistent with experimental results, and the possible reaction pathways for CO_2_RR to CO on N, P co‐doped carbon are shown in Figure 5b. Furthermore, the significant decrease in free energy barrier for *COOH adsorption was confirmed by N, P co‐doping. The free energy barrier of 0.81 eV on model NPC−C was much lower than that of 1.64 eV on model NC−C, indicating that the presence of P atoms stabilized the key *COOH intermediate. Interestingly, the rate‐determining step (RDS) moved to the second electron transfer step on model NPC−C. Based on experimental results, the formation of *COOH is the most important step for CO generation.

According to the previous literature, the formation of *COOH is a key step for CO_2_‐to‐CO reduction, and the CO generation can be inhibited by the high Gibbs free energy of *COOH adsorption.[^40^, ^41^] The free energy diagram at −0.8 V vs. RHE reveals that the effect of N and P co‐doping is more remarkable (Figure 5c). There is no change in RDS between 0 V and −0.8 V vs. RHE, but the free energy barrier for RDS was reduced extremely on model NPC−C. In addition to CO_2_RR, the free energy diagrams for HER were also calculated on model NC−C and NPC−C. The notable difference was observed between N doping and N, P co‐doping at 0 V vs. RHE (Figure S14a). The RDS on model NC was the adsorption of *H, while the RDS on NPC was the desorption of *H. However, the free energy barrier for RDS was almost the same between the two models. Even at −0.8 V vs. RHE, every free energy diagram presented a downhill, and there is no difference in the HER activity between N doping and N, P co‐doping (Figure S14b). Overall, introduced N atoms serve the preferable CO_2_ adsorption sites, and the P atoms present near the N atoms stabilize the *COOH key intermediates, leading to promoting CO_2_RR to CO.

## Conclusions

Metal‐free N, P co‐doped carbons (NPCs) were synthesized with various N and P contents by tuning the raw material composition. NPCs generated the syngas, and the ratio (H_2_/CO) can be controlled within the high demand range (0.3–4). Experimental analysis and density functional theory (DFT) calculation reveal that negatively charged C atoms adjacent to N and P atoms are the most favorable active sites for CO_2_RR to CO on N, P co‐doped carbon. Furthermore, the co‐doping of N and P atoms decreases the Gibbs free energy barrier for key *COOH intermediates adsorbed on C atoms. Our work provides not only the rational design of N, P co‐doped carbons to control the syngas ratio, but also the deep insight into the reaction mechanisms for CO_2_RR to CO on N, P co‐doped carbons.

## Conflict of Interests

The authors declare no conflict of interest.

1

## Supporting information

As a service to our authors and readers, this journal provides supporting information supplied by the authors. Such materials are peer reviewed and may be re‐organized for online delivery, but are not copy‐edited or typeset. Technical support issues arising from supporting information (other than missing files) should be addressed to the authors.

Supporting Information

## Data Availability

The data that support the findings of this study are available from the corresponding author upon reasonable request.
