# Supplementary material for: Metal‐Free N, P‐Codoped Carbon for Syngas Production with Tunable Composition via CO2 Electrolysis: Addressing the Competition Between CO2 Reduction and H2 Evolution
Source: ChemSusChem. 2025 Jan 3;18(7):e202402249. doi: 10.1002/cssc.202402249 (PMC11960591; doi:10.1002/cssc.202402249)
Supplement: Supplementary file 1 — Supporting Information [file CSSC-18-e202402249-s001.pdf]

ChemSusChem

Supporting Information

Metal-Free N, P-Codoped Carbon for Syngas Production with Tunable Composition via CO_2 Electrolysis: Addressing the Competition Between CO_2 Reduction and H_2 Evolution

Ryuji Takada,* Hiroyuki Okada, Kotaro Narimatsu, Koji Miyake,* Yoshiaki Uchida, Etsushi Tsuji, and Norikazu Nishiyama

Metal-free N, P-Codoped Carbon for Syngas Production with Tunable Composition via CO_2 Electrolysis: Addressing the Competition Between CO_2 Reduction and H_2 Evolution

Ryuji Takada,*[a] Hiroyuki Okada,[b] Kotaro Narimatsu,[a] Koji Miyake,*[a, c] Yoshiaki Uchida,[a] Etsushi Tsuji,[b] Norikazu Nishiyama[a, c]

[a] Mr. R. Takada,* Mr. K. Narimatsu, Dr. K. Miyake,* Prof. Y. Uchida, Prof. N. Nishiyama
Division of Chemical Engineering, Department of Materials Engineering Science
Graduate School of Engineering Science, Osaka University
1-3 Machikaneyama, Toyonaka, Osaka 560-8531, Japan
E-mail: r.takada@cheng.es.osaka-u.ac.jp, kojimiyake@cheng.es.osaka-u.ac.jp

[b] Mr. H. Okada, Prof. E. Tsuji
Center for Research on Green Sustainable Chemistry, Tottori University
4-101 Koyama, Tottori, Tottori 680-0945, Japan

[c] Dr. K. Miyake, Prof. N. Nishiyama
Innovative Catalysis Science Division, Institute for Open and Transdisciplinary Research Initiatives (ICS-OTRI), Osaka University
Suita, Osaka 565-0871, Japan

Experimental Section

***Materials*:** Glycine, aqueous solutions of 50 wt.% phytic acid, and Nafion (5 wt.%) were purchased from FUJIFILM Wako Pure Chemical (Japan). N_2 gas (purity 99.99%) and CO_2 gas (purity 99.5%) were purchased from Iwatani (Japan). All the materials were used as received without further purification.

***Synthesis procedure*:** Glycine and phytic acid in a mass ratio of 1: x (x = 0.5, 1, 2) were dissolved in deionized water, and the solution was placed in a ceramic boat and dried at 90 °C. After that, the dried solid materials were carbonized for 3 h at 1100 °C under an N_2 atmosphere in tubular furnace. The obtained carbon catalysts were denoted NPC-x. The same carbonization process was performed using only glycine or phytic acid, with the products denoted NC and PC, respectively. To obtain the porous N doped carbon, NC-act was synthesized by activated NC with CO_2 for 3 h 950 ºC.

***Characterizations*:** Transmission electron microscope (TEM) and scanning electron microscope-energy dispersive X-ray spectrometry (SEM-EDX) were respectively performed using an H800 (Hitachi, Japan) and a JCM-7000 (JEOL, Japan). X-ray diffraction (XRD) was carried out on a PANalytical X'Pert-MDR diffractometer using Cu Kα radiation. Raman spectra of samples were recorded using a confocal Raman microscope (LabRAM HR-800, Horiba, Ltd., Kyoto, Japan). X-ray photoelectron spectroscopic (XPS) was performed using a Kratos Ultra 2 (Shimadzu, Japan), and the N_2 adsorption isotherms were measured at –196.15 °C using a BELSORP MINI X (Microtrac MRB, Japan). Accordingly, the specific surface areas and pore volumes and size distributions were determined using the BET, Barrett-Joyner-Halenda and MP-Plot methods. Organic elemental analysis was conducted using FlashEA (Thermo Fisher Scientific, USA).

***Preparation of the working electrodes*:** Nafion (5 wt.% in alcohol and water), isopropanol, and water were mixed to prepare the catalyst dispersion solution in a volume ratio of 1:1:8. This solution (520 mL) was added to the catalyst (3 mg) and ultrasonicated to prepare the catalyst ink. Subsequently, the ink (30 μL) was pipette onto a carbon paper three times, yielding a mass loading of 1 mg·cm^{-2}.

***Electrochemical measurements*:** All the electrochemical measurements were performed using a Biologic SP-50e potentiostat (TOYO, Japan) with an Ag/AgCl (3 M KCl solution) as a reference electrode, a platinum wire as a counter electrode, and an as-

prepared working electrode at a H-type cell. The potentials in this study are relative to the RHE, based on the following equations:

$$E_{RHE} = E_{Ag/AgCl} + 0.208 + 0.0591 \times \text{pH}$$

Linear sweep voltametric (LSV) measurements were performed in CO_2-saturated and N_2-saturated 0.1 M $KHCO_3$ solution with a scan rate of 5 mV s^{-1}, and the potential was applied from 0.15 V to -1.2 V vs. RHE. During the LSV measurements, CO_2 and N_2 are kept flowing.

CO_2 splitting experiments were carried out in CO_2-saturated 0.1 M $KHCO_3$ solution with stirring at 350 rpm. Before CO_2 splitting experiments, the 0.1 M $KHCO_3$ solution was pre-saturated with CO_2 for at least 30 minutes. Gas products were analyzed by an off-line gas chromatography (GC-80A, Shimazu, Japan) with a Shincarbon-ST column (SHINWA, Japan). The liquid products were analyzed using an off-line high-performance liquid chromatograph (EXTREMA, JASCO, Japan) with a Unifinepak C18 04250-5M column (JASCO, Japan) and a RSpak KC-811 (Shodex, Germany). The faradaic efficiencies of the gas products were calculated by the following equations:

$$FE = \frac{moles\ of\ products}{Q/nF} \times 100\%$$

FE (%) = faradaic efficiency;
Q (C) = electric quantity;
n (−) = electron transfer number;
F (C/mol) = Avogadro constant, 96485.33 C/mol

Computational details: B3YLYP hybrid density functional theory (DFT) of Gaussian 16W[S1] was performed with a basis set of 6-31G (d, p) to obtain the stabilized model, the atomic population, and the various thermodynamic energy. Several possible heteroatoms doped carbon configurations, containing pyridinic N and P-C bond, were used as calculated model in this study. The ground state structures of *COOH, *CO, and *H adsorbed on heteroatoms doped carbon model were determined by searching the lowest energy one among all the possible configurations on pyridinic N and C atoms adjacent to N and P atoms as possible active sites.

The adsorption energy was calculated by the following equation:

$$E_{ads} = E_{complex} - E_{substrate} - E_{adsorbate}$$

where E_{ads}, $E_{complex}$, $E_{substrate}$, and $E_{adsorbate}$ are the adsorption energy of adsorbate (*COOH, *CO, *H) on substrate (heteroatoms doped carbon), the total energy of adsorbate on substrate, the total energy of substrate, and the total energy of adsorbate.

According to computational hydrogen electrode (CHE) model, the Gibbs free energy was calculated by following equation[S2]:

$$\Delta G = \Delta E_{ads} + \Delta E_{ZPE} - T\Delta S$$

Where ΔE_{ads} is different adsorption energy of adsorbate, ΔE_{ZPE} is zero-point energy changes calculated by the vibration frequency of each adsorbate, T is temperature (298.15 K), and ΔS is entropy change of the reaction.

In this work, the reaction pathway of CO_2 electroreduction into CO was based on following elementary step:

$$* + \mathrm{CO_2(g)} + \mathrm{H^+} + \mathrm{e^-} \rightarrow \mathrm{COOH^*}$$

$$\mathrm{COOH^*} + \mathrm{H^+} + \mathrm{e^-} \rightarrow \mathrm{CO^*} + \mathrm{H_2O(l)}$$

$$\mathrm{CO^*} \rightarrow * + \mathrm{CO(g)}$$

The reaction pathway of HER was based on following elementary step:

$$* + \mathrm{H^+(aq)} + \mathrm{e^-} \rightarrow \mathrm{H^*}$$

$$\mathrm{H^*} + \mathrm{H^+(aq)} + \mathrm{e^-} \rightarrow * + \mathrm{H_2}(g)$$

The * indicates the active site on substrate.

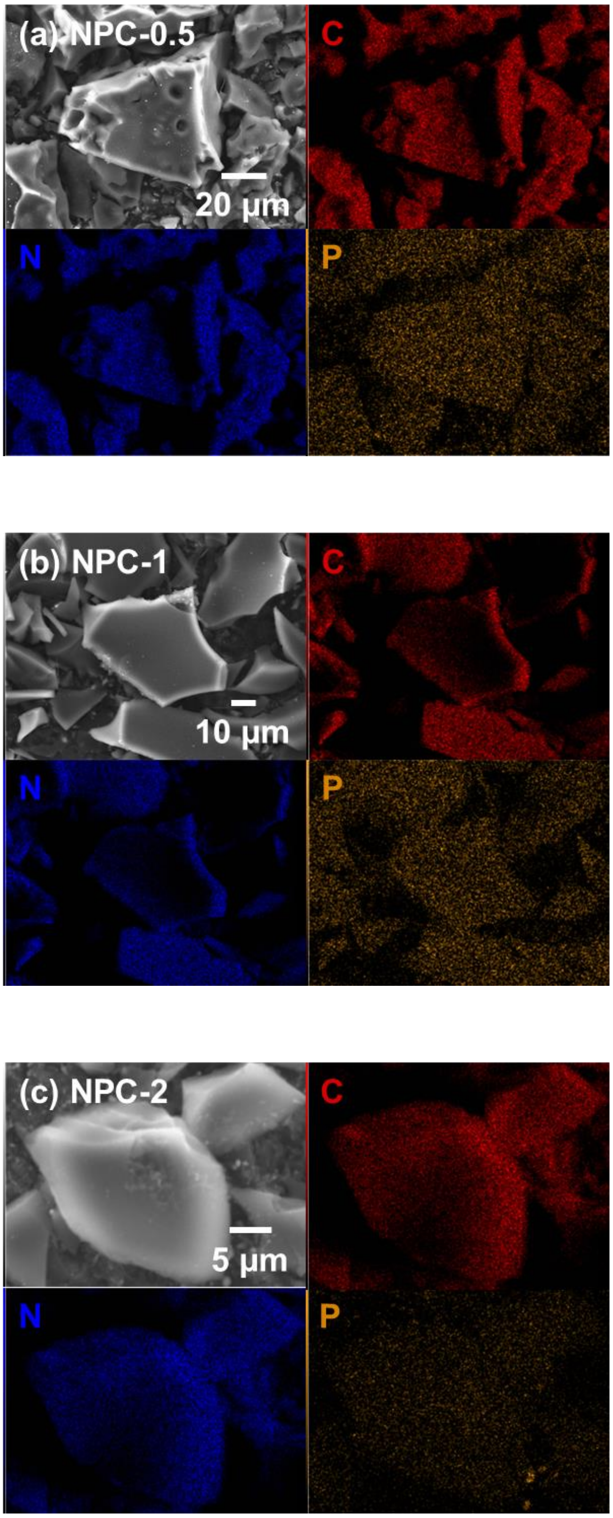

Figure S1. Elemental mapping images of a) NPC-0.5, b) NPC-1, and c) NPC-2.

Table S1. The contents of C, N, P, and O in each catalyst.

Catalysts	C [wt %]	N [wt %]	P [wt %]	O [wt %]
NC	89.80	3.50	–	6.24
NPC-0.5	81.61	2.50	3.81	11.13
NPC-1	82.23	2.34	3.66	11.22
NPC-2	77.42	1.81	6.75	13.12
PC	74.14	–	6.21	19.18
NC-act	84.98	3.52	–	10.40

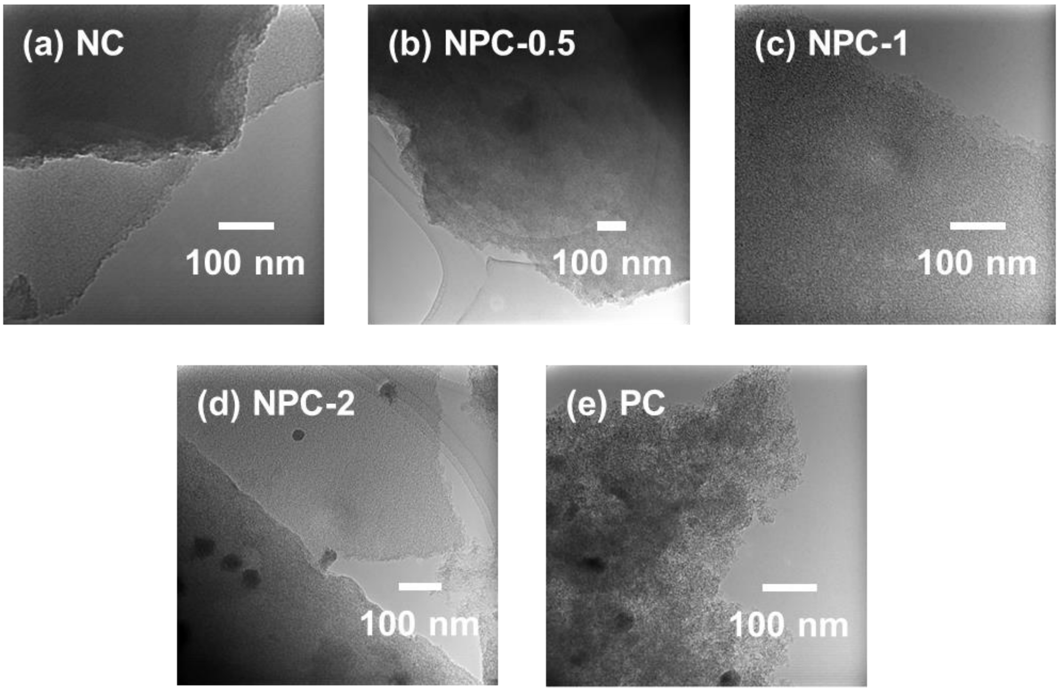

Figure S2. TEM images of a) NC, b) NPC-0.5, c) NPC-1, d) NPC-2, and e) PC.

Table S2. BET surface area and pore volume of each pore region of each catalyst.

Sample	S_{BET} [m^2/g]	V_{total} [cm^3/g]	V_{macro} [cm^3/g]	V_{meso} [cm^3/g]	V_{micro} [cm^3/g]
NC	200	0.1450	0.0164	0.0313	0.0973
NPC-0.5	841	0.4085	0.0134	0.0171	0.3780
NPC-1	1201	0.6488	0.0262	0.0662	0.5564
NPC-2	1169	0.7542	0.0652	0.1550	0.5340
PC	1420	1.4691	0.3761	0.6638	0.4292
NC-act	1140	0.8789	0.0116	0.2374	0.6299

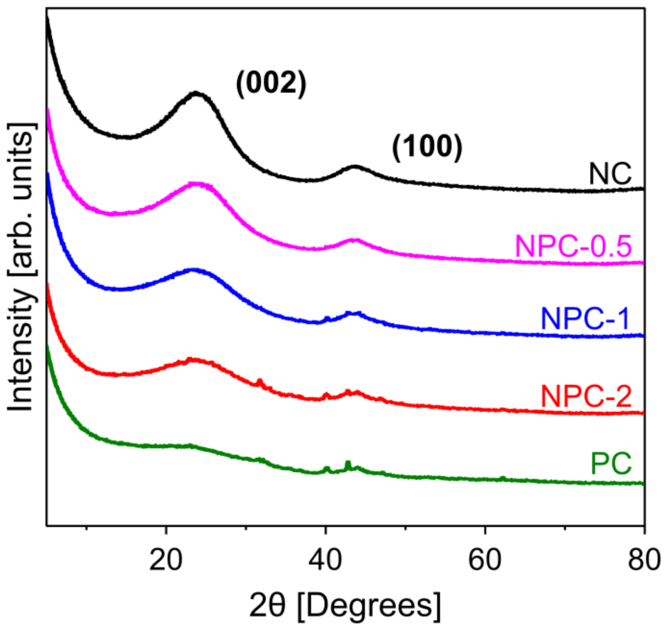

Figure S3. XRD patterns of each catalyst.

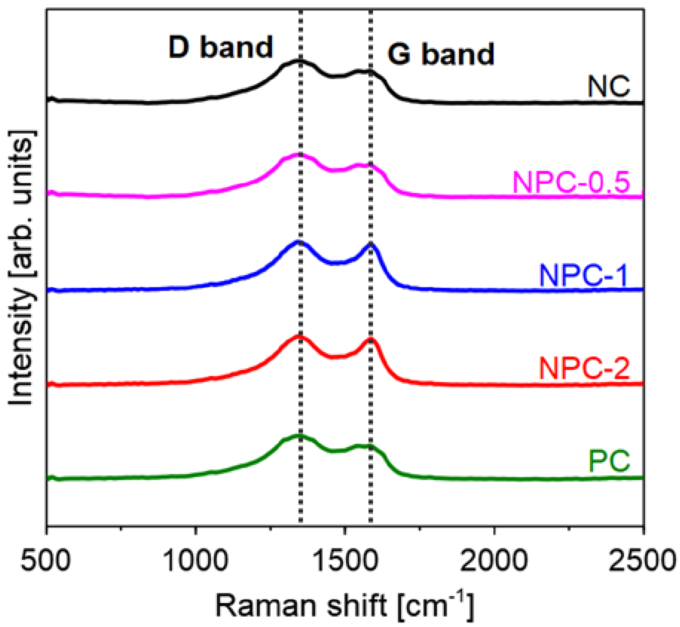

Figure S4. Raman spectra of each catalyst.

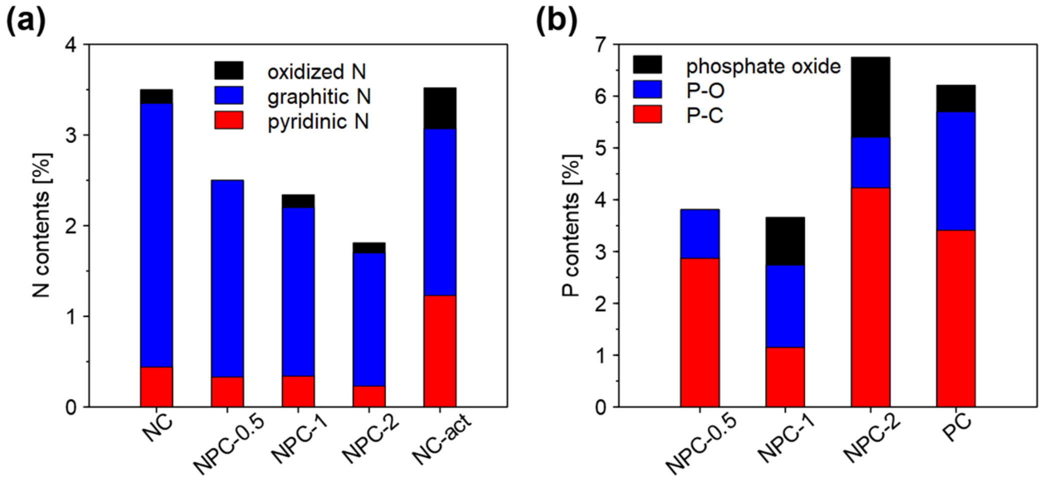

Figure S5. The contents of (a) each N species, and (b) each P species.

Table S3. Faradaic efficiencies of CO and H_2 at -0.8 V vs. RHE using each catalyst in CO_2-saturated 0.1 M $KHCO_3$ solution.

Sample	FE_{CO} [%]	FE_{H2} [%]
NC	32.3	45.2
NPC-0.5	45.3	45.3
NPC-1	56.0	40.8
NPC-2	70.4	26.8
PC	24.8	57.8
NC-act	55.8	29.7

Table S4. Faradaic efficiencies of gas products using NPCs within the potential window from -0.6 V to -1.2 V vs. RHE in CO_2-saturated 0.1 M $KHCO_3$ solution and the molar ratio of H_2 and CO.

Sample	Potential [V]	FE_{CO} [%]	FE_{H2} [%]	H_2/CO [-]
NPC-0.5	-0.6	55.8	30.5	0.6
	-0.8	45.3	45.3	1.0
	-1.0	20.6	79.5	3.9
	-1.2	6.4	94.0	14.7
NPC-1	-0.6	57.3	29.9	0.5
	-0.8	57.7	40.3	0.7
	-1.0	21.9	78.5	3.6
	-1.2	8.9	90.2	10.1
NPC-2	-0.6	63.6	21.8	0.3
	-0.8	70.4	26.8	0.4
	-1.0	46.3	52.1	1.1
	-1.2	10.3	89.1	8.7

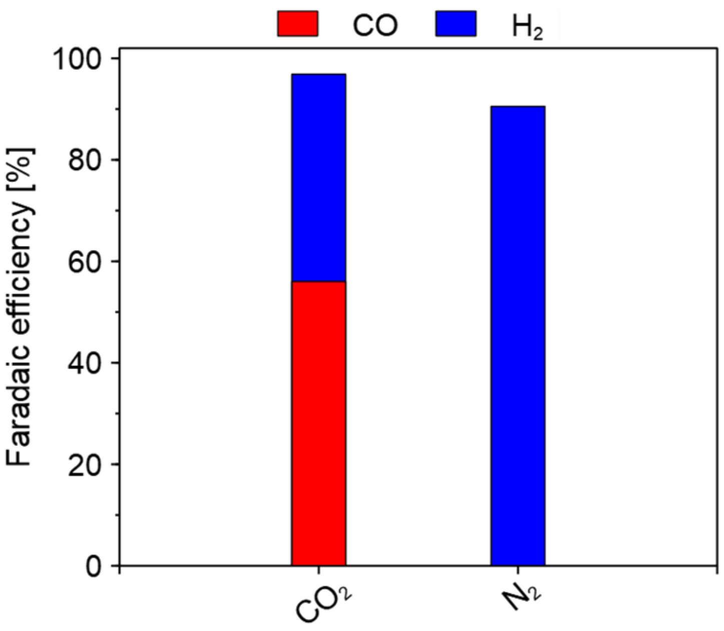

Figure S6. Faradaic efficiency using NPC-1 at -0.8 V vs. RHE in CO_2- or N_2- saturated 0.1 M $KHCO_3$ solution.

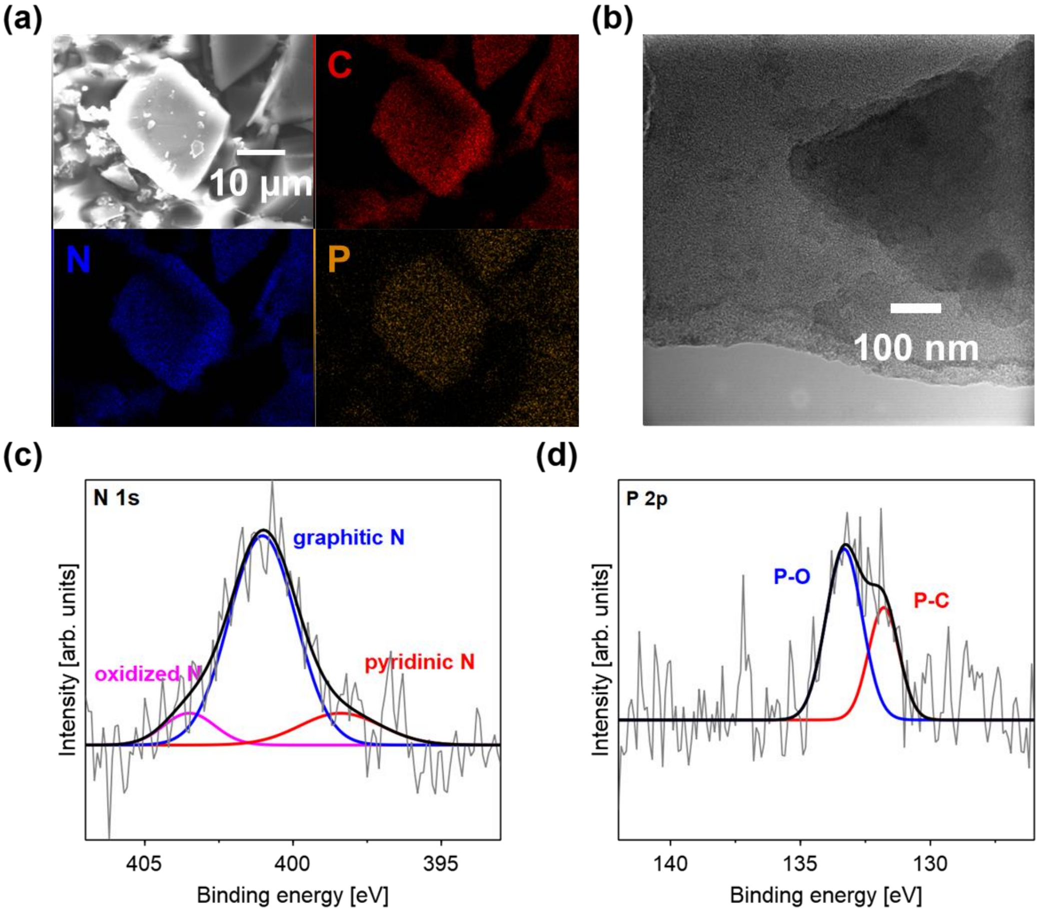

Figure S7. a) Elemental mapping image, b) TEM image, and XPS spectra of NPC-1 after long-term test: c) N 1s, and d) P 2p.

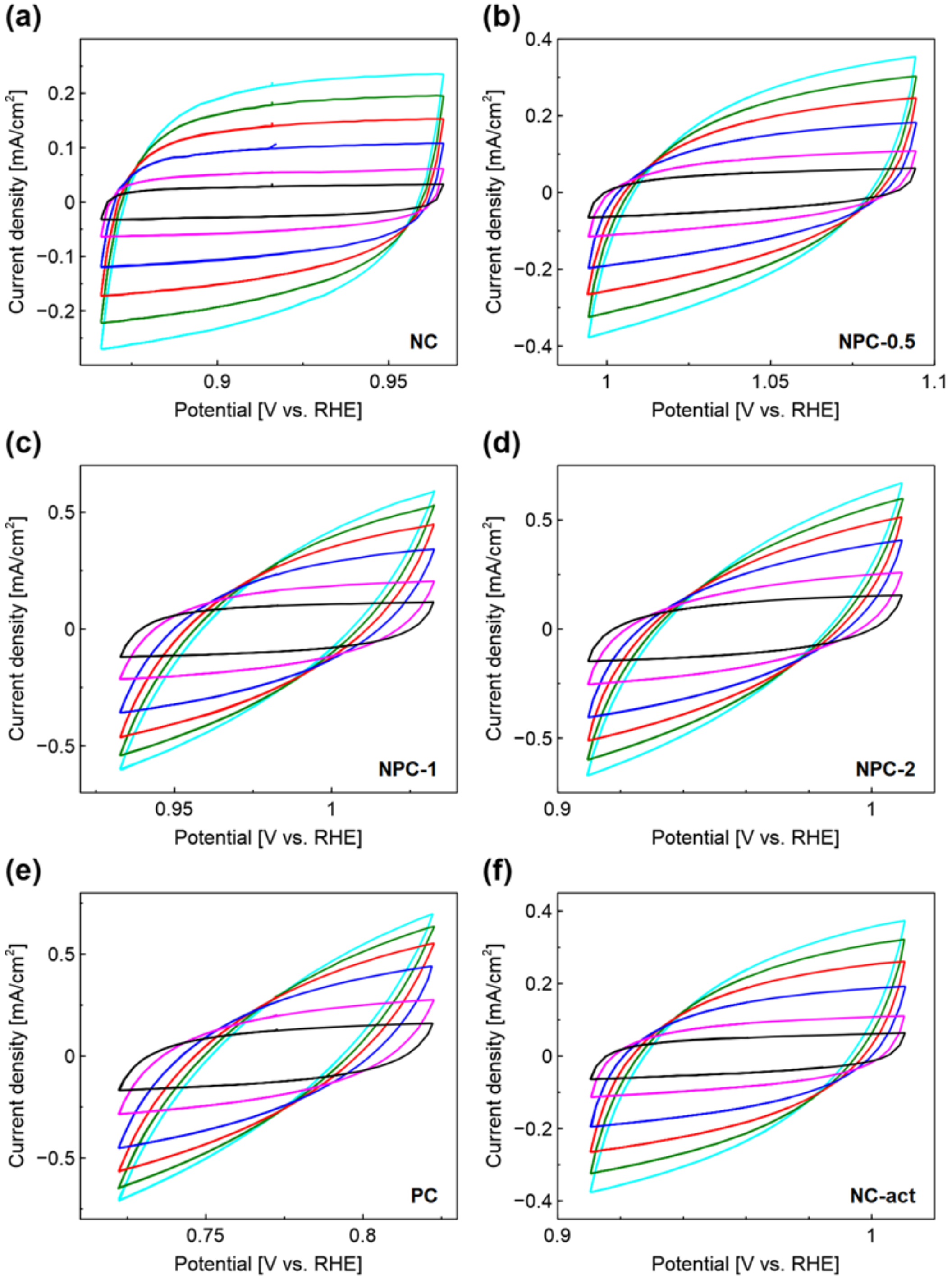

Figure S8. Cyclic voltammograms of each catalyst in 0.1 M $KHCO_3$ at various scan rate for estimation of double layer capacitance (C_{dl}). The scan rate: 5, 10, 20, 30, 40, and 50 mV/s.

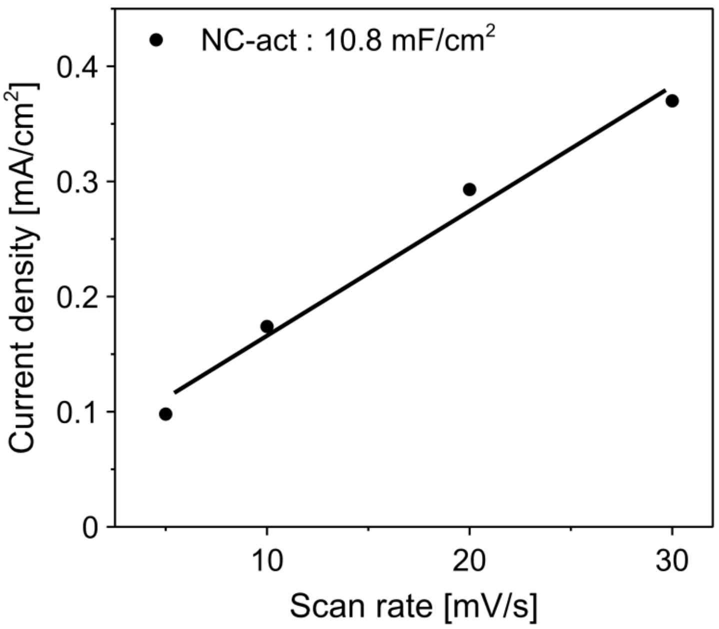

Figure S9. The C_{dl} value of NC-act.

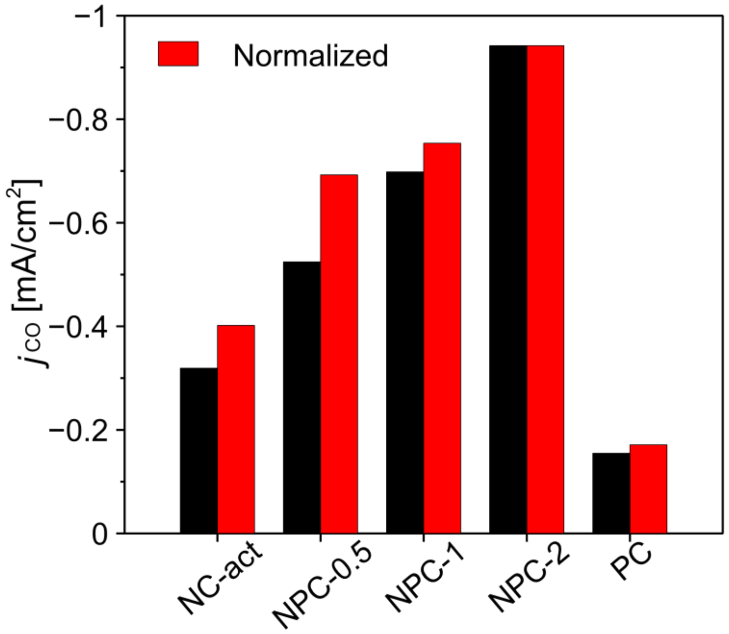

Figure S10. The comparison partial current density of CO (j_{CO}; black bar) and the j_{CO} normalized by ECSA (normalized j_{CO}; red bar) of each catalyst at -0.8 V vs. RHE.

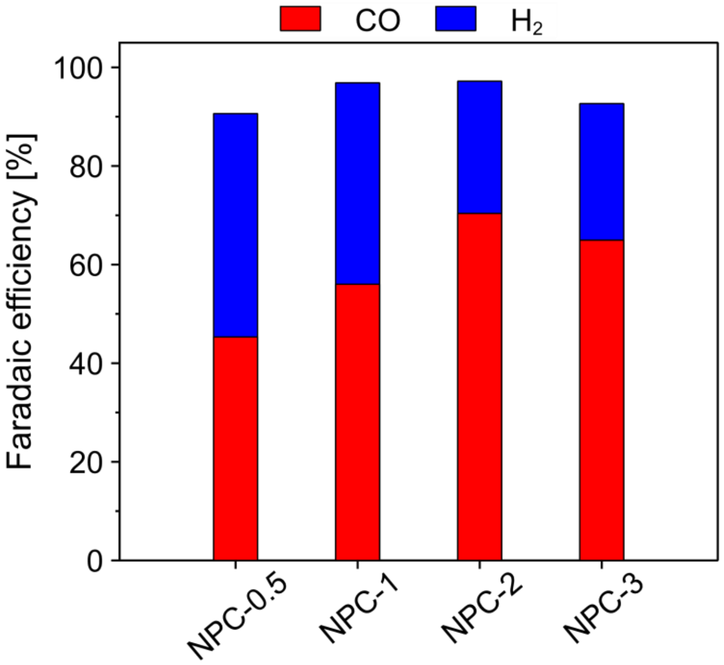

Figure S11. Faradaic efficiency using NPC-*x* (*x* = 0.5, 1, 2, 3) at -0.8 V vs. RHE in CO_2-saturated 0.1 M $KHCO_3$ solution.

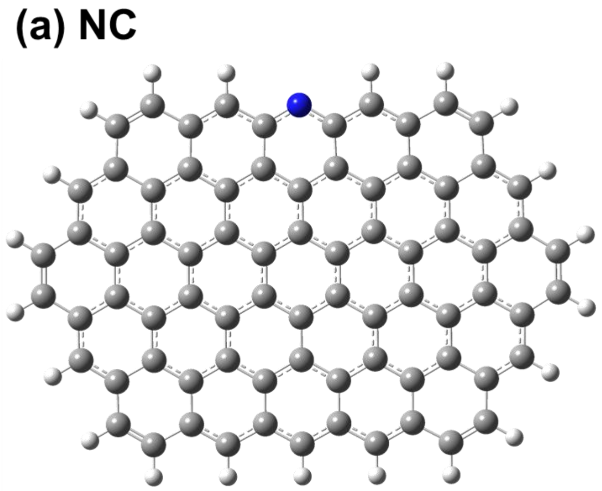

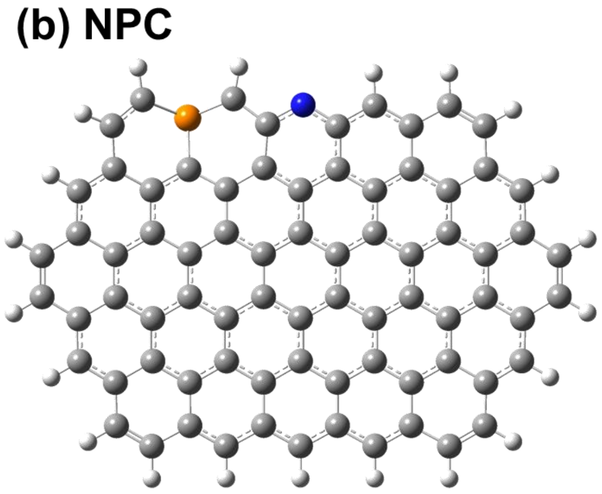

Figure S12. The computational structure models of (a) N doped carbon and (b) N, P co-doped carbon configurations.

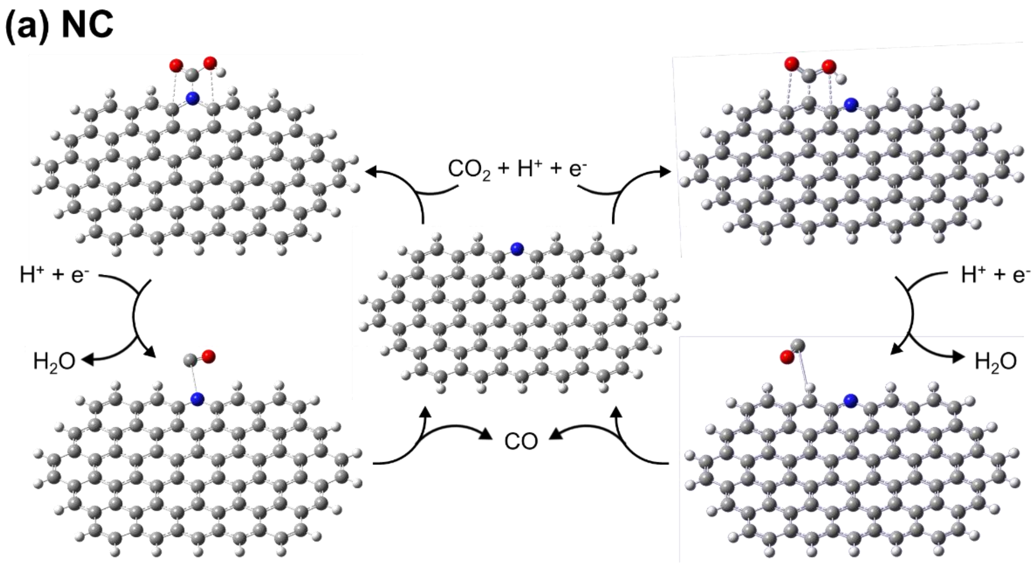

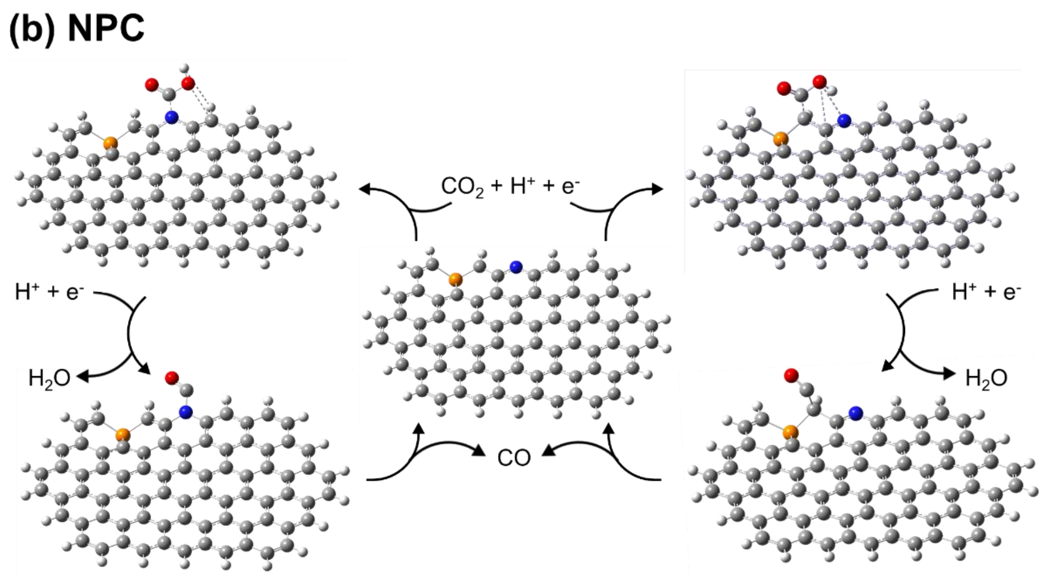

Figure S13. Reaction pathways for CO_2RR to CO on a) model NC, and b) model NPC.

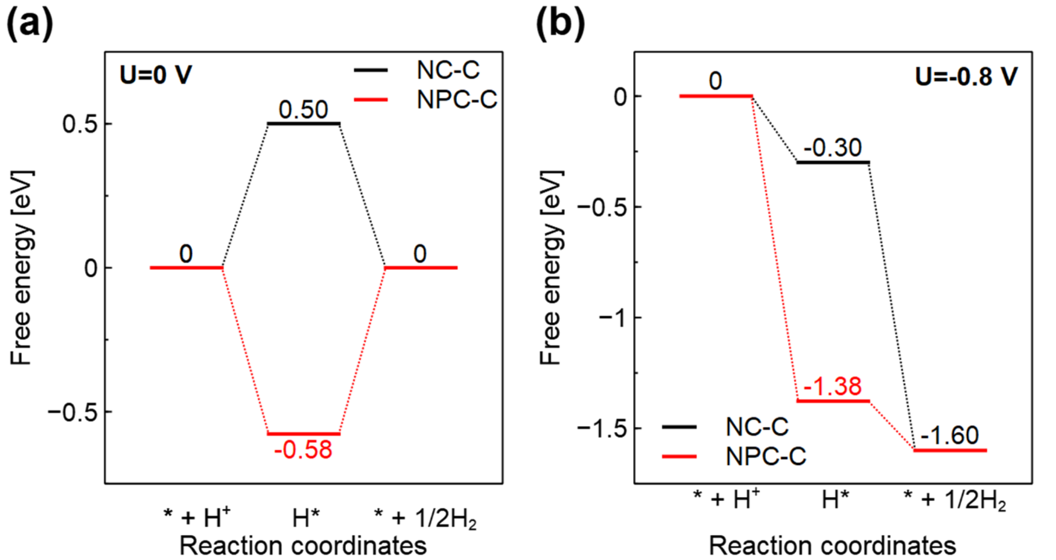

Figure S14. Gibbs free energy diagrams for HER on model NC-C and NPC-C.
a) U=0 V. b) U=-0.8 V.

References

[S1] Frisch, M. J.; Trucks, G. W.; Schlegel, H. B.; Scuseria, G. E.; Robb, M. A.; Cheeseman, J. R.; Scalmani, G.; Barone, V.; Petersson, G. A.; Nakatsuji, H.; Li, X.; Caricato, M.; Marenich, A. V.; Bloino, J.; Janesko, B. G.; Gomperts, R.; Mennucci, B.; Hratchian, H. P.; Ortiz, J. V.; Izmaylov, A. F.; Sonnenberg, J. L.; Williams, F.; Ding, F.; Lipparini, F.; Egidi, J.; Goings, B.; Peng, A.; Petrone, T.; Henderson, D.; Ranasinghe, V. G.; Zakrzewski, J.; Gao, N.; Rega, G. Z.; Liang, W.; Hada, M.; Ehara, M.; Toyota, K.; Fukuda, R.; Hasegawa, J.; Ishida, M.; Nakajima, T.; Honda, Y.; Kitao, O.; Nakai, H.; Vreven, T.; Throssell, K.; Montgomery, J. A. Jr.; Peralta, J. E.; Ogliaro, F.; Bearpark, M. J.; Heyd, J. J.; Brothers, E. N.; Kudin, K. N.; Staroverov, V. N.; Keith, T. A.; Kobayashi, R.; Normand, J.; Raghavachari, K.; Rendell, A. P.; Burant, J. C.; Iyengar, S. S.; Tomasi, J.; Cossi, M.; Millam, J. M.; Klene, M.; Adamo, C.; Cammi, R.; Ochterski, J. W.; Martin, R. L.; Morokuma, K.; Farkas, O.; Foresman, J. B.; Fox, D. J. Gaussian16 Rev. C. 01, Wallingford, CT, 2016. C. Lee, W. Yang, R. G. Parr, Phys. Rev. B **1988**, *37*, 785–789.

[S2] A. A. Peterson, F. Abild-Pedersen, F. Studt, J. Rossmeisl, J. K. Nørskov, *Energy Environ. Sci.* **2010**, *3*, 1311.
